# Annexin A2 Acts as an Adhesion Molecule on the Endometrial Epithelium during Implantation in Mice

**DOI:** 10.1371/journal.pone.0139506

**Published:** 2015-10-07

**Authors:** Bing Wang, Tian-Min Ye, Kai-Fai Lee, Philip C. N. Chiu, Ronald T. K. Pang, Ernest H. Y. Ng, William S. B. Yeung

**Affiliations:** 1 Department of Obstetrics and Gynaecology, The University of Hong Kong, Pokfulam Road, Hong Kong, People’s Republic of China; 2 Key Laboratory of Medical Reprogramming Technology, Shenzhen Second People’s Hospital, First Affiliated Hospital of Shenzhen University, Shenzhen, People’s Republic of China; 3 Shenzhen Key Laboratory of Fertility Regulation, The University of Hong Kong-Shenzhen Hospital, Shenzhen, People’s Republic of China; 4 Centre for Reproduction, Development and Growth, The University of Hong Kong, Pokfulam Road, Hong Kong, People’s Republic of China; South China Agricultural University, CHINA

## Abstract

To determine the function of Annexin A2 (Axna2) in mouse embryo implantation in vivo, experimental manipulation of Axna2 activities was performed in mouse endometrial tissue in vivo and in vitro. Histological examination of endometrial tissues was performed throughout the reproduction cycle and after steroid treatment. Embryo implantation was determined after blockage of the Axna2 activities by siRNA or anti-Axna2 antibody. The expression of Axna2 immunoreactivies in the endometrial luminal epithelium changed cyclically in the estrus cycle and was upregulated by estrogen. After nidatory estrogen surge, there was a concentration of Axna2 immunoreactivities at the interface between the implanting embryo and the luminal epithelium. The phenomenon was likely to be induced by the implanting embryos as no such concentration of signal was observed in the inter-implantation sites and in pseudopregnancy. Knockdown of Axna2 by siRNA reduced attachment of mouse blastocysts onto endometrial tissues in vitro. Consistently, the number of implantation sites was significantly reduced after infusion of anti-Axna2 antibody into the uterine cavity. Steroids and embryos modulate the expression of Axna2 in the endometrial epithelium. Axna2 may function as an adhesion molecule during embryo implantation in mice.

## Introduction

Embryo implantation consists of 3 tightly regulated events, namely apposition, adhesion and penetration. In mice, apposition occurs in the afternoon of Day 4 of pregnancy, attachment begins at around 10–11:00 pm of the same day, and invasion starts in the morning of the next day [1–3]. Although implantation is crucial to reproduction, the molecules involved in the process are not fully known and is a hot research area.

To address the question, we determined the differentially regulated surface proteins on receptive (4 pm of Day 4 of pregnancy) endometrial luminal epithelium (LE) relative to non-receptive (Day 1 of pregnancy) LE in mice using biotin labeling followed by 2-dimensional gel electrophoresis and tandem mass spectrometry. Our unpublished results showed a 2-fold increase in the expression of annexin A2 (Axna2) in the mouse receptive LE, a result similar to that in human endometrium [4–6]. AXAN2 was recently identified as one of the apical surface molecules in a receptive human endometrial cell line [7].

Axna2 is a member of the annexin family. Annexins are proteins that bind to anionic phospholipids in a calcium dependent manner. At least 12 annexins are found in higher vertebrates [8]. Membrane bound Axna2 is a heterotetramer consisting of two Axna2 and two S100A10 (S100 calcium binding protein A10) molecules. Axna2 is a substrate of Src (Rous sarcoma oncogene) protein kinase [9], and is involved in cellular transformation, differentiation [10], regulation of secretory process, prolactin release and prostaglandin formation [8].

Axna2 was studied recently in the field of reproduction. In human endometrium, the expression of Axna2 is high in the receptive phase [6] and is low in the pre-receptive phase [4, 5], consistent with a role of Anxa2 in implantation. Dysregulation of interleukin 11 expression in endometrium is associated with infertility probably via action of the cytokine on blastocyst-endometrial epithelium adhesion [11]. The cytokine upregulates the expression of Axna2 in the primary human endometrial epithelial cells and an endometrial cell line [12]. Using in vitro human models, ANXA2 is linked to the RhoA/ROCK pathway, which in turns affects trophoblast adhesiveness on endometrial cells, migration of endometrial epithelial cells and outgrowth of trophoblast [13]. The relevance of these studies to implantation in vivo is not known. We hypothesized that Anxa2 was involved in implantation. The present study intended to explore the function of Axna2 in mouse embryo implantation in vivo. The results show that estradiol (E2) controls Axna2 expression in the LE of mice, and that the implanting embryos modulate the expression of Axna2 at the implantation sites. Our data also provide the first direct evidence on the involvement of Axna2 in implantation in vivo.

## Materials and Methods

### Animals

All procedures for handling of animals were approved by the Committee on the Use of Live Animal in Teaching and Research, the University of Hong Kong. Bilateral ovariectomy was performed on 6-week old mice under anesthesia. The success of the operation was confirmed by daily vaginal smears for 4 days after a 4 week of clearing period. The ovariectomized mice were injected subcutaneously with either equal volume of vehicle (sesame oil), 100 ng/mouse of E2, 1 mg/mouse of P4, or a combination of 100 ng/mouse of E2 and 1 mg/mouse of P4 as described [14] to produce circulating steroid levels similar to that in the estrus cycle. The mouse uteri were collected 24 hour later for immunohistochemical staining. Euthanasia was performed by overdose administration of pentobarbital.

Pseudopregnant mice were prepared by allowing mating of the nulliparous mature female ICR mice with vasectomized males. The day of the presence of a vaginal plug was defined as Day 1 of pseudopregnancy. Mated female mice were housed individually before experimentation.

### In vivo knockdown of Axna2

Fresh siRNA-liposome complexes were prepared by mixing 20 μl of siRNA solution (Thermo Scientific, NY, USA) containing 80 or 160 pmol siRNA with 20 μl of Lipofectamine 2000 solution (Invitrogen, Carlsbad, USA) immediately before each experiment. After incubation for 15 minutes at room temperature, 20 μl of the preparation was injected into the lumen of the uterine horns of Day 3 pseudopregnant mice. On Day 4 of pseudopregnancy, the mice were euthanatized and the uterine horns were collected. The siRNA was applied to Day 3 psuedopregnant uterus to allow time for the siRNA to exert its knockdown action.

### Infusion of anti-Axna2 antibody into uterus

Antibody was prepared by mixing 50 μl of anti-Axna2 antibody (1 mg/ml, ab41803, Abcam, Cambridge. UK) or normal rabbit IgG with 1 ml of PBS and spinning of the mixture through an Amicon Ultra–15 Centrifugal Filter Unit with Ultracel–10 membrane (Millipore, MA, USA) at 4°C to remove preservative in the antibody/IgG solution. Ten microliters of the buffer-exchanged anti-Axna2 antibody (10 μg) per uterine horn was infused into pregnant mice on Day 4 of pregnancy between 4:00 and 6:00 pm. The same volume of buffer-exchanged IgG with the same protein concentration was infused into the contralateral horn as control. Mice were killed 2 days later and the numbers of implantation site were counted.

### Endometrial tissue culture model for implantation

The endometrial tissue implantation model and the antibody treatment procedure were reported elsewhere [15]. In brief, Day 4 blastocysts were placed on isolated endometrial tissue of Day–4 pregnant mice and cocultured for 28 hours. The number of blastocysts that remained attached after a brief washing was counted.

### Immunostaining of Axna2

Uteri from pseudopregnant and pregnant mice at 4:00 pm of Day 1, 10:00 am, 4:00 pm, 11:00 pm of Day 4, 10:00 am, 4:00 pm and 11:00 pm of Day 5, and 10:00 am of Day 6 of pregnancy were collected, fixed, embedded in paraffin wax and sectioned. For collection of uteri at different estrous stages, vaginal smears were checked before tissue collection. For immunohistochemical staining, slides were blocked in 10% goat serum for 1 hour at room temperature before incubation with 0.5 μg/ml anti-Axna2 antibody overnight at 4°C. The slides were then successively incubated with 0.5 μg/ml of goat anti-rabbit secondary biotinylated antibody (Dako, Glostrup, Denmark) for 40 minutes, Vectastain elite ABC reagents (Vector Laboratories, Burlingame, CA, USA) for 30 minutes, and 3’ diaminobenzidine tetrahydrochloride (DAB, Dako) for 1–3 minutes. To better observe the expression of Axna2 on plasma membrane, the immunofluorescence technique was used. For immunofluorescence staining, sections were incubated with 1:1000 AlexFluor–488 labeled secondary antibody (Invitrogen, Carlsbad, US) for 1 hour. All incubations with the primary antibody were done at room temperature. Each batch of samples were processed and developed in exactly the same conditions. Images of the stained sections were captured under a fluorescence microscope (Eclipse Ti, Nikon Tokyo, Japan). IgG control and anti-Axna2 antibody preabsorbed control showed no signal in the staining. Besides, mouse colon and liver were used as positive and negative control, respectively in the optimization experiment.

### H-scoring

For each slide, one image at 200 x magnification was taken to show the general annexin A2 intensity in the uterine LE, glandular epithelium (GE) and stroma. Another 10–15 images at 1000x magnification were taken randomly for H-Scoring (histological scoring) of the intensities of Axna2 immunoreactivities in the LE, GE and stroma. All the images were ranked by another colleague of the laboratory having no relationship with the project. The scores were calculated according to the equation H-SCORE = ΣPi (i+1), where i = intensity that ranges from 0 (no signal) to 3 (strong signal), and Pi = percentage of cells.

### Western blot analysis

Endometrial tissues were frozen in liquid nitrogen and grinded into powder. The proteins in the powder were dissolved in RIPA lysis buffer (RIPA: 1X PBS, 1% Nonidet P–40, 0.5% sodium deoxycholate, 0.1% SDS), resolved in 10% SDS gel electrophoresis, and transferred to PVDF membrane (Millipore, Temecula, MA, USA). The membrane was blocked with 5% skimmed milk before incubation with primary anti-Axna2 antibody overnight at 4°C. The membrane was then washed in phosphate buffered saline containing Tween 20 (PBST) five times for 5 minutes each, incubated with 1:5000 horseradish peroxidase-conjugated secondary antibody (Sigma, Castle Hill, NSW, Australia) with shaking for 1 hour at room temperature. After washing five times with PBST for 5 minutes each, specific signal on the membrane was detected on X-ray film (Galen, UK) by WESTSAVE Up^TM^ enhanced chemiluminescent solution (Abfrontier Co. Ltd., Seoul, Korea) according to the manufacturer’s protocol. The membrane was stripped with mild reblot solution (Millipore) and reprobed with alpha tubulin (Abcam) which served as an internal control. The gel image was scanned and analyzed by the Image Pro Plus (Media Cybernetics, MD, US).

### Statistical analysis

One Way Analysis of Variance on rank was used to compare the quantitative data, which were presented as median and ranges. The data on attachment rates in the in vitro model were expressed as percentage attached blastocysts relative to the number of seeded blastocysts for co-culture. Statistical comparisons were performed using Chi square test. A difference with P<0.05 was considered to be statistically significant.

## Results

### Expression of Axna2 in estrus cycle and early pregnancy

**Fig 1:**
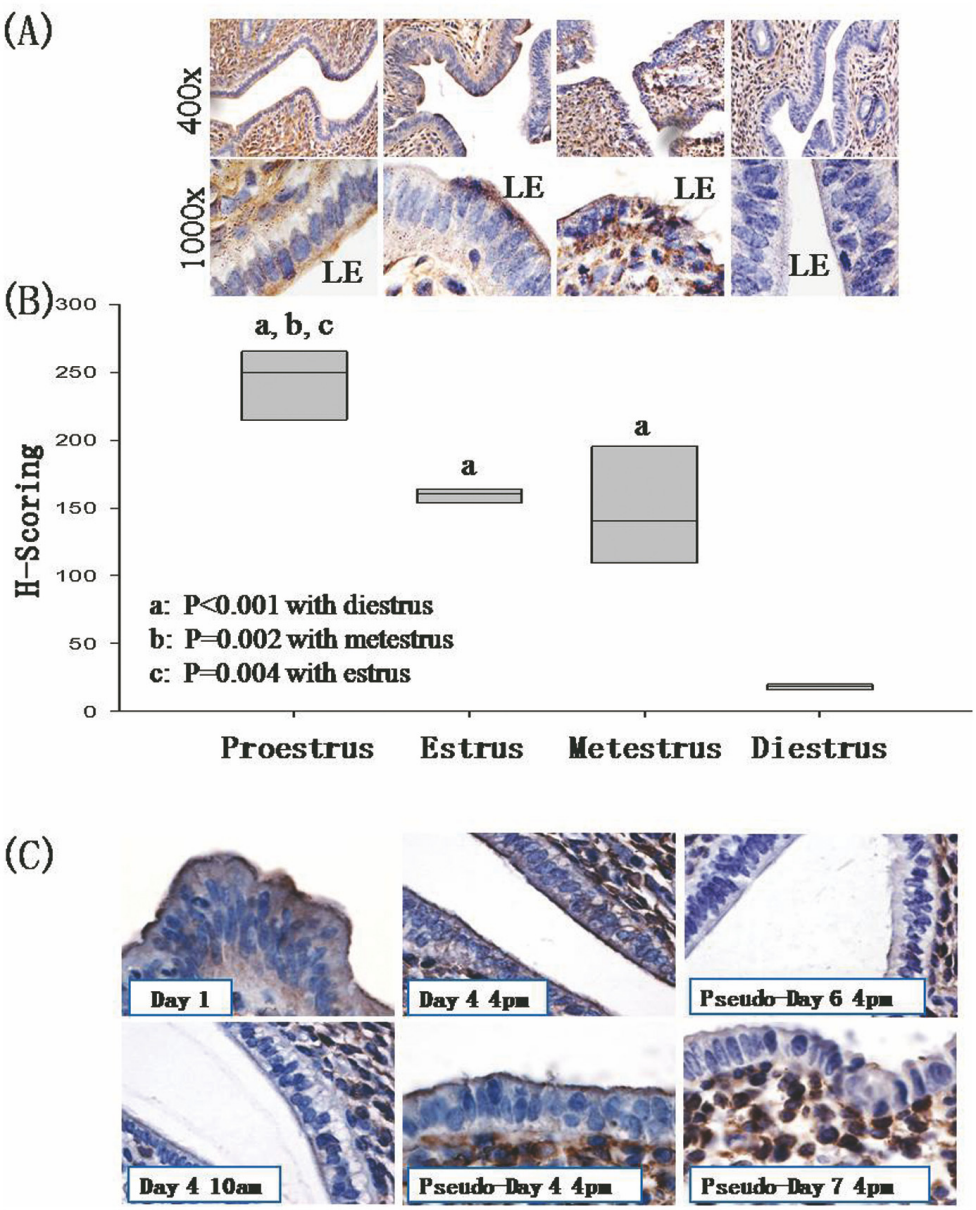
Axna2 expression in mouse endometrial luminal epithelium. **(A)** Endometrial Axna2 expression in the estrous cycle: Axna2 expression was high in the luminal epithelium (LE) at proestrous, and absent at diestrous. **(B)** H-Scoring of the immunostaining. ^a^ P<0.001 with diestrus, ^b^ P = 0.002 with metestrus. ^c^ P = 0.004 with estrus. (**C)** Axna2 expression in mouse LE in early pregnancy. Axna2 expression was increased after estradiol surge (10:00 am—12 noon) on Day 4 of pregnancy, and was decreased on Day 6 and Day 7 of pregnancy. Magnification: 1000x.

There was an increase in Axna2 immunoreactivities on the apical surface of the LE from proestrus to estrus stage (Fig 1A). The intensity of the staining decreased at metestrus stage. No Axna2 immunoreactivities were detected at diestrus stage. H-Scoring confirmed the observed expression pattern semi-quantitatively (Fig 1B). A significant lower score (P<0.05) was found in the diestrus stage when compared with the other stages. Proestrus stage had the highest signal intensities.

The immunohistochemical study of Axna2 was also conducted in mice from Day 1 to 7 of pregnancy. Axna2 immunoreactivities were expressed on the apical surface of LE on Day 1 of pregnancy (Fig 1C). The signal was hardly detected in the LE at 10:00 am on Day 4 of pregnancy. The expression of Axna2 on the apical membrane of LE was greatly increased at 4:00 pm after nidatory estrogen surge, and the intensities of the staining were comparable to that in the LE at 4:00 pm on Day 4 of pseudopregnancy. The signal intensity in the LE decreased greatly after Day 5 and was undetectable by Day 6 of pseudopregnancy.

### Expression of Axna2 at the implantation sites

**Fig 2:**
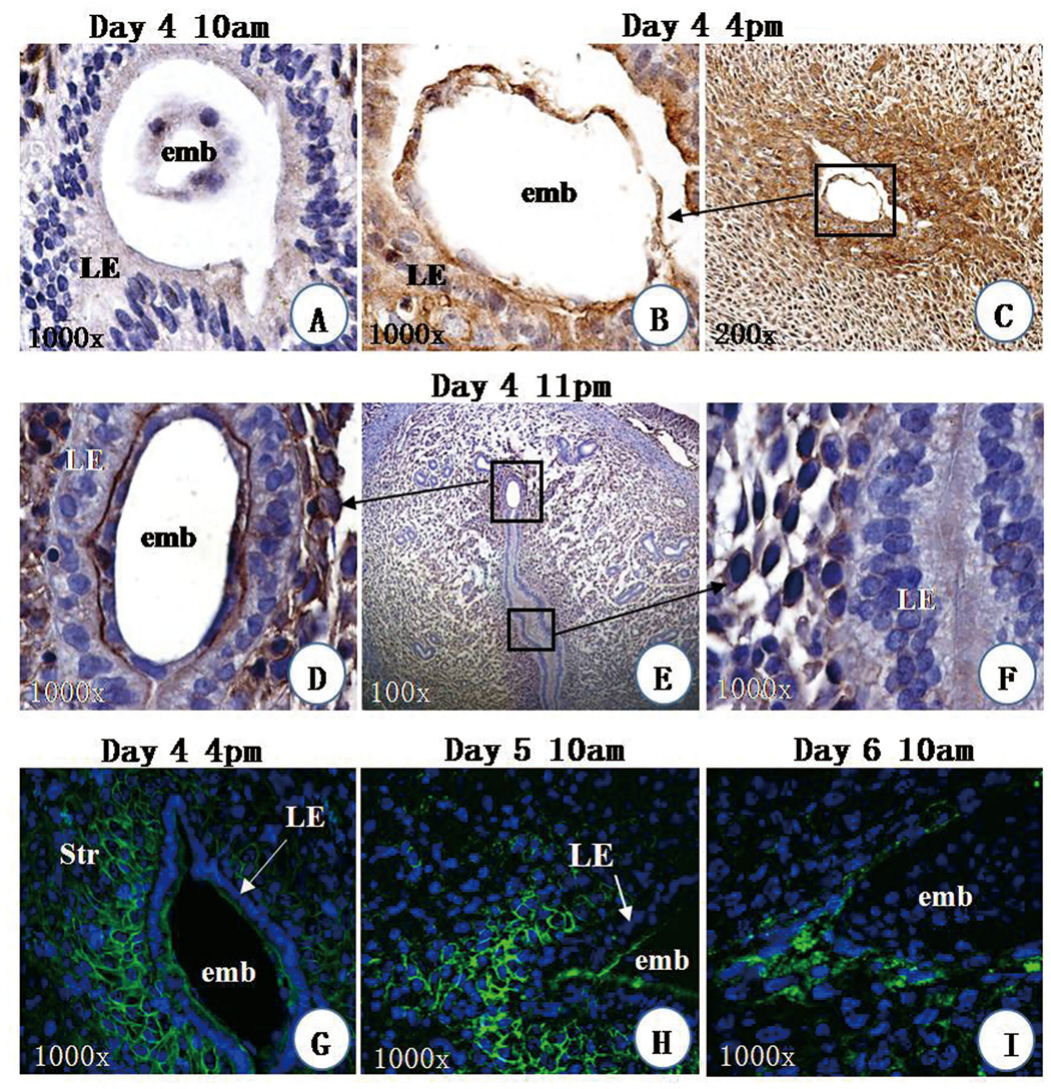
Expression of Axna2 at implantation sites. On Day 4 of pregnancy, the immunoreactivities (brown) at the implantation sites were weak at 10:00 am (**A)**. The signal increased at 4:00 pm **(B).** At 4:00 pm, the signal was strong at the implantation sites **(B)** and in the nearby stroma (Str) (**C**). Concentration of signal was observed at the interface between the luminal epithelium (LE) and the embryo (emb) **(D, E)** but not between LE **(F)**. Immunofluorescence staining showed that Axna2 immunoreactivies (green) were on the membrane of the endometrial stroma on Day 4 **(G)** and Day 5 **(H)** of pregnancy. The immunoreactivies became cytosolic on Day 6 of pregnancy **(I).**

To study the possible action of embryos on expression of Axna2, immunostaining was performed at the implantation site. The Axna2 immunoreactivities of the LE next to the implanting embryos were weak at 10:00 am on Day 4 of pregnancy (Fig 2A). The signal in the LE increased at 4:00 pm (Fig 2B). Interestingly, the immunoreactivities of the implanting embryos also increased. The signal became strong and concentrated at the embryo-LE interface at 11:00 pm on the same day (Fig 2D and 2E) and not between the LE (Fig 2F). No such concentration of signal was observed at the inter-implantation site (data not shown). The strong signal at the implantation site lasted throughout Day 5 and decreased by Day 6 of pregnancy.

Fig 2G–2I show the expression of Axna2 in the endometrial stroma at the implantation site using immunofluorescence staining. Axna2 was strongly expressed on the membrane of the subluminal stromal cells closely beneath the implanting embryo from 4:00 pm of Day 4 (Fig 2C and 2G) and Day 5 of pregnancy (Fig 2H), but was relocated to the cytosol on Day 6 of pregnancy (Fig 2I).

### Steroids regulate expression of Axna2 in the mouse endometrial LE

**Fig 3:**
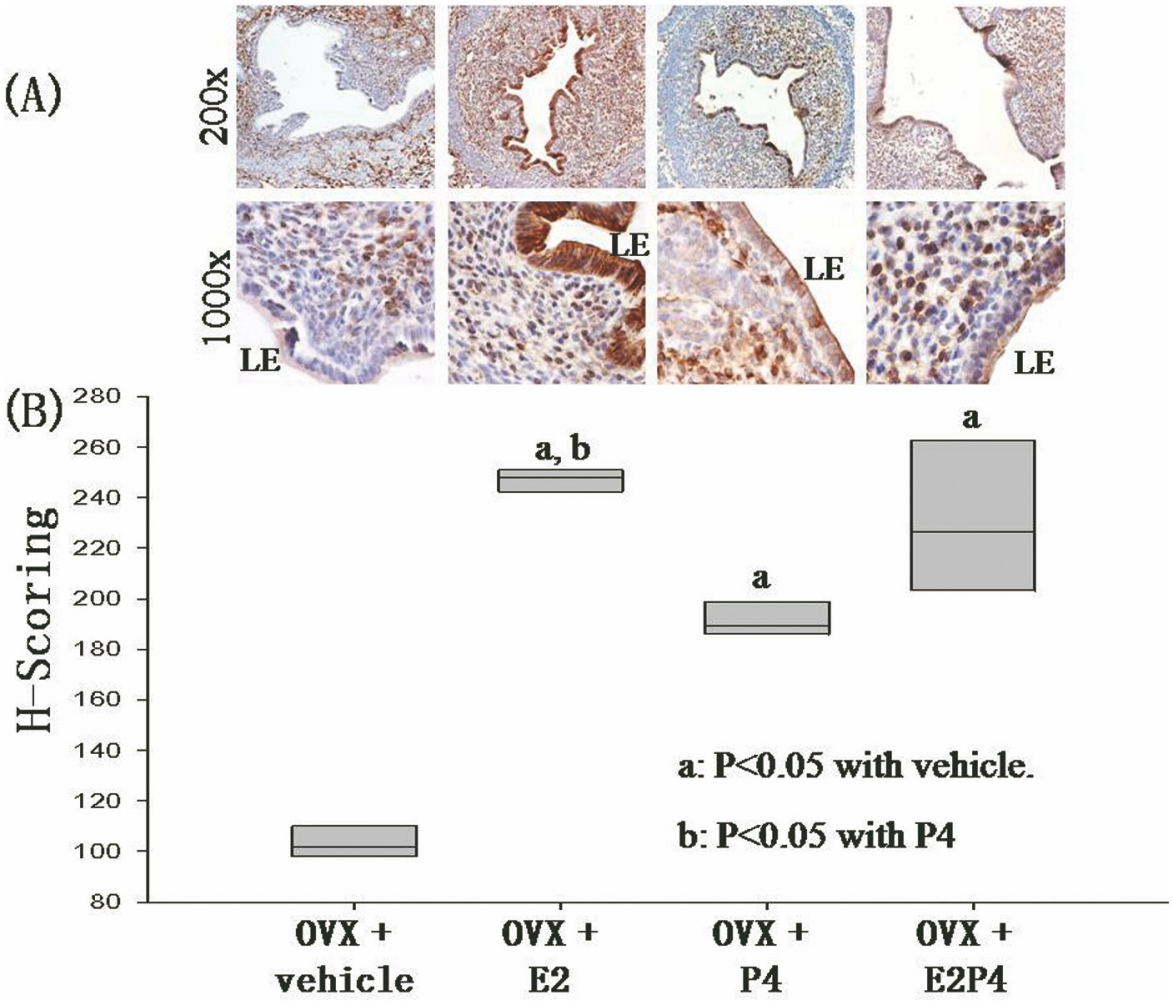
Effects of estradiol and progesterone on the expression of Axna2 in endometrial luminal epithelium of ovariectomized mice. **(A)** The expression of Axna2 was increased by estradiol (E2), progesterone (P4) and their combined (E2P4) treatment. E2 had the most potent effect. **(B)** H-Scoring of immunohistochemical staining. ^a^ P<0.05 with control (ctrl), ^b^ P<0.05 with P4.

To study steroid regulation of Axna2, ovariectomized mice were treated with vehicle, estradiol (E2), progesterone (P4), or combined estradiol and progesterone (E2P4). The expression of Axna2 was low in the ovariectomized mice. E2, P4, and E2P4 treatment increased the expression of Axna2 (Fig 3A). Strong Axna2 immunoreactivities were observed mainly in the cytoplasm of LE after E2 treatment. Treatment with P4 and E2P4 also increased the Axna2 immunoreactivities with a significant portion of the signal concentrated on the apical surface of LE. Fig 3B shows the H-scoring of Axna2 intensities on the LE upon steroid treatments. All the three treatments significantly increased the scores when compared with that in the ovariectomized mice. E2 treatment showed the strongest effect and had a score significantly higher than that after P4 treatment.

### Knockdown of Axna2 suppresses blastocyst attachment in endometrial tissue implantation model

To study the role of Axna2 in implantation, an in vivo knockdown approach by siRNA was used. Two different doses of siRNA were tested. Western blotting revealed that the higher dose (160 pmol) of siRNA reduced the Axna2 expression level by about 43% in the transfected endometrium when compared with the siRNA control and untreated Day 4 endometrium of pseudopregnant mice (S1 Fig). Immunostaining showed that the epithelium and stroma of the knockdown tissue has reduced expression of Axna2 (S2 Fig).

**Fig 4:**
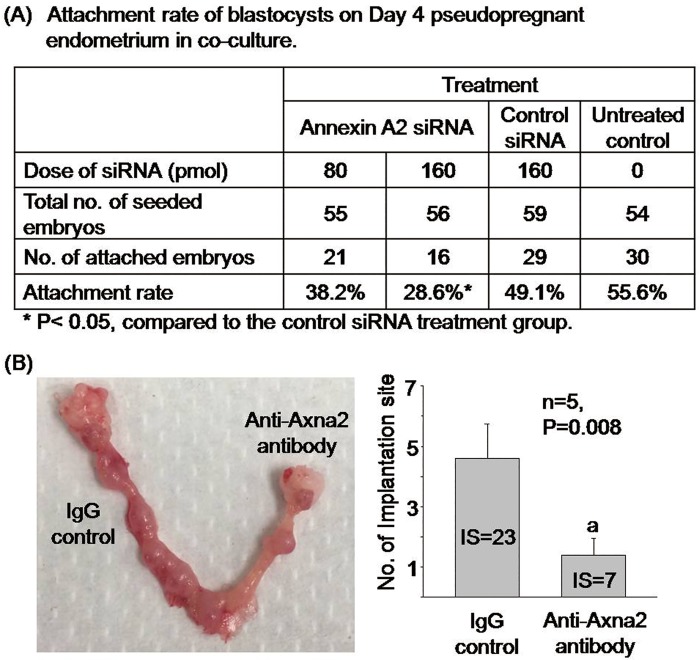
Effect of Axna2 on implantation. **(A)** Attachment rate of mouse blastocysts in an endometrial tissue culture model for implantation. The attachment rate on Day 4 pseudopregnant endometrium was significantly suppressed after transfection of the endometrium with Axna2 siRNA. **(B)** The number of implantation sites (IS) was significantly decreased after uterine infusion of anti-Axna2 antibody. Right: 10 μl of normal IgG; Left: 10 μl of anti-Axna2 antibody. Bar chart shows the number of implantation sites in the anti-Axna2 antibody and IgG treated uteri. P = 0.008 between the two groups (n = 5).

Blastocysts attachment assays on the endometrial tissues transfected with Axna2 siRNA/scramble control were performed. The blastocyst attachment rates on the endometrium transfected with 80 pmol and 160 pmol of Axna2 siRNA were 38.2% and 28.6%, respectively (Fig 4A). The attachment rate on the endometrium transfected with control siRNA was 49.1% while that on untreated endometrium (Day 4 pseudopregnant mice) was 55.6%. There was a significant difference (p<0.05) in the attachment rate between the 160 pmol Axna2 siRNA transfected groups and the control groups. Although the attachment rate of the 80 pmol siRNA Axna2 was reduced when compared to the 2 control groups, statistical significant differences were not yet reached (P>0.05).

### Surface membrane associated Axna2 is involved in the attachment of embryo to endometrium

For in vivo functional study, 10 μl of buffer exchanged anti-Axna2 antibody was infused into one uterine horn of Day 4 pregnant mice at 4:00–6:00 pm. The same volume and concentration of buffer exchanged IgG was infused in the contralateral horn as control. As shown in Fig 4B, the implantation sites was reduced by 3-fold (P = 0.008) after anti-Axna2 antibody infusion.

## Discussion

While there are some in vitro data using cell lines to represent embryo and LE during implantation supporting a role of Axna2 in human implantation, the regulation of Axna2 during implantation in vivo is not fully known. This study showed that steroids regulate the expression of Axna2 in the mouse endometrial LE. More importantly, the results showed for the first time that the implanting embryos modulate the expression of Axna2 and that the outer surface membrane-bound Axna2 was involved in the attachment of embryos onto the endometrium in vivo.

### Steroids regulate mouse endometrial luminal epithelial expression of Axna2

In the mouse estrous cycle, the cyclical pattern of Axna2 in the endometrial LE coincided with that of E2. The Axna2 immunoreactivities were high on the apical surface of LE from proestrous to estrus stage and after nidatory E2 surge. The high LE expression of Axna2 immunoreactivities the next morning (Day 1 of pregnancy) probably represented the time lag required for turnover of the Axna2 protein produced at estrus. The close link between E2 and Axna2 was likely due to an action of E2 on Axna2 expression as the phenomenon also occurred in pseudopregnancy, which had the same hormonal profile though without the implanting embryos. After implantation, the expression of Axna2 in the LE of Day 6 pseudopregnant mice was reduced consistent with the low E2 level at the time.

The action of E2 on the expression of Axna2 was confirmed in the ovariectomized mice model. It was noted that Axna2 immunoreactivities were uniformly distributed in the cytosol of LE in the E2 treated group, but were mainly on the apical membrane of LE in the P4 and E2P4 treated groups. The difference was likely due to the action of P4 on the secretory activities of LE, and Axna2 is known to be involved in cellular exocytosis [16–18] and Axna2 can be a secretory protein [19].

Bioinformatics analysis of the mouse Axna2 gene supports a direct action of E2 on Axna2 expression. Although no consensus estrogen responsive element (ERE) (GGTCAnnnTGACC) is found in the promoter of mouse Axna2, there is a cluster of 5 closely located half-ERE binding site (TGACC) separated by 18–174 base pairs found from -4117bp to -3870 bp and a number of other variations of the consensus ERE (see S1 Table) within -4000 bp. Half binding sites and ERE variations have been shown to be responsive to estrogen–estrogen receptor complex (reviewed by ref [20]). It has to be confirmed by gene reporter assays whether the half-ERE sites in the Axna2 promoter of mouse are functional.

The mouse Axna2 promoter region also contains glucocorticoid-responsive element (GRE) [21]. Given the highly conserved structure of GRE and progesterone responsive element, the mouse Axna2 gene is likely responding directly to P4 treatment as well.

### Implanting embryos modulate mouse endometrial luminal epithelial expression of Axna2

The relationship between Axna2 and implanting embryos was investigated by comparing the LE expression of Axna2 at the implantation sites and at the inter-implantation sites. Axna2 expression increased sharply upon embryo attachment and became concentrated at the interface between the embryo and the LE from Day 4 4:00 pm till Day 5 11:00 pm. No such concentration of Axna2 signal was found at the inter-implantation site and at the implantation sites away from the implanting embryos and after penetration. The observations indicated modulatory action of the embryo on Axna2 expression in the early phase of implantation, and suggested that Axna2 was mobilized to the plasma membrane upon intimate contact of the embryo with the LE. It is not known how this is accomplished and can only be speculated upon.

Calcium is implicated in embryo implantation. Infusion of calcium channel blocker (diltiazem) into the mouse uterine cavity on Day 4 of pregnancy causes complete implantation failure [22, 23]. Upon attachment, intracellular calcium of the embryo and the LE may increase through calcium influx or intracellular calcium pool release. There was an increased expression of Axna2 mRNA from the morula to the blastocyst stage in mice and pigs [24], and Axna2 protein was also expressed in a bovine trophectoderm cell line [25] and a human trophoblast cell line (Wang B, unpublished data). It has been demonstrated that ErbB4 on mouse embryos mediates endometrial heparin-binding EGF-like growth factor (HB-EGF)-induced calcium influx of mouse trophoblast [26], and that ligation of integrin elevates mouse trophoblast intracellular calcium levels through calcium influx [27, 28]. Although there is no evidence demonstrating that the same happens in the LE, an in vitro implantation model using an endometrial epithelial cell line (RL95-2) and trophoblast (JAr) spheroids as embryo surrogates demonstrates increase in intracellular calcium of the RL95-2 cells after attachment of the spheroids onto the cells [29]. Given that the membrane association and translocation of Axna2 was calcium dependent, the cell contact may induce Axna2 relocation through the action of calcium elevation.

Another phenomenon was noted at the implantation site: Axna2 was strongly expressed and adopted a membrane-bound form in the stromal cell during invasion at the implantation site. The phenomenon may be related to calcium elevation during decidualization because deciduogenic concanavalin A-loaded beads can induce a higher level of calcium in the decidualized regions than in the non-decidualized regions [30]. After implantation, not only the intensity of Axna2 in the stroma decreased, its distribution also changed from membrane bound back to cytosol. The observed expression pattern of Axna2 was similar to that of COX2 [3, 31], suggesting a possible role of the Axna2 in decidualization.

### Axna2 and embryo implantation

Four observations suggested that Anxa2 functioned as an adhesion molecule during implantation. First, Anxa2 is located on the apical surface of the mouse endometrium. Similar observation is also found in humans[7]. Second, there was a strong expression of Axna2 at the embryo-LE interface. Third, intrauterine knockdown of Axna2 in pseudopregnant mice decreased the attachment of the blastocyst onto the treated endometrial tissue in vitro. Fourth, infusion of anti-Axna2 antibody into the uterine horn led to a 3-fold decrease in the number of implantation sites in vivo.

This is the first report on impairment of embryo implantation by blockage of Axna2 in the mouse LE during the implantation window. The results are consistent with a previous report showing that knockdown of S100A10, the binding partner of Axna2 heterotetramers, caused implantation failure in mice [32].

Axna2 is involved in cell adhesion in other systems. Axna2-S100A11 serves as a molecular bridge for cell-cell adhesion between breast cancer cells and microvascular endothelial cells [33]. Apart from adhesion, invasion of the trophoblast through the endometrial epithelium is an integral process of implantation, involving migration of the trophoblast cells. Tyrosine phosphorylation of Axna2 regulates morphological changes associated with cell motility via Rho-mediated actin rearrangement [10].

In sum, this study revealed a novel function of Axna2 during implantation in mice. It is likely that Axna2 is involved in human implantation as well. ANXA2 is expressed mainly in the luminal epithelium of human mid- and late-secretory endometria [13]. The expression of the molecule is dramatically diminished in the endometrial epithelial cells in the presence of intrauterine devices [4]. In vitro study also suggests a role of Axna2 in embryo adhesiveness [13]. Whether its abnormal expression on LE is related to subfertility needs further investigation.

## Supporting Information

S1 Fig Supplementary Fig 1 Knockdown of Axna2 in mouse endometrium. Western blotting was performed on the mouse endometrium transfected with and without Axna2 siRNA. The expression of Axna2 of endometrium transfected with 160 pmol Axna2 siRNA was lower than those transfected with control siRNA and freshly collected endometrium from Day 4 pseudopregnant mice.
(TIF)

S2 Fig Supplementary Fig 2 Immunohistochemical staining of annexin A2 on endometrium transfected with siRNA. The annexin A2 expression level in the endometrium transfected with 160 pmol annexin A2 siRNA (B) was lower than that transfected with 160 pmol scramble control siRNA (C) and the freshly dissected Day 4 pseudopregnant mice endometrium (D). While the annexin A2 expression level in the endometrium transfected with 80 pmol annexin A2 siRNA (A) was not much lower than the controls. LE: Luminal epithelium; GE: Glandular Epithelium; SC: Stromal cells. Magnification ×400.
(TIF)

S1 File The raw data for Figs 1 & 3 and S1 Fig. (XLSX)

S1 Table Supplementary Table 1. (DOCX)
