# Supplementary figures and images for: Annexin A2 Acts as an Adhesion Molecule on the Endometrial Epithelium during Implantation in Mice
Source: PLoS One. 2015 Oct 7;10(10):e0139506. doi: 10.1371/journal.pone.0139506 (PMC4596619; doi:10.1371/journal.pone.0139506)

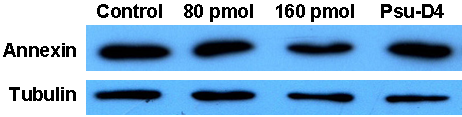

Supplement: S1 Fig — Western blotting was performed on the mouse endometrium transfected with and without Axna2 siRNA. The expression of Axna2 of endometrium transfected with 160 pmol Axna2 siRNA was lower than those transfected with control siRNA and freshly collected endometrium from Day 4 pseudopregnant mice. (TIF) [file pone.0139506.s001.tif]

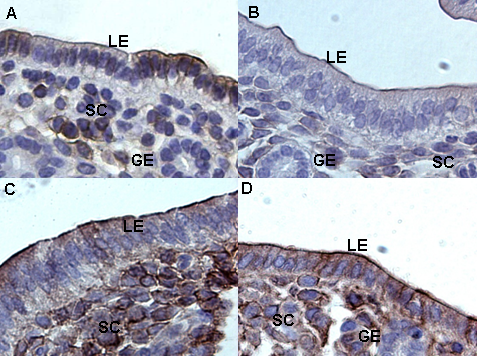

Supplement: S2 Fig — The annexin A2 expression level in the endometrium transfected with 160 pmol annexin A2 siRNA (B) was lower than that transfected with 160 pmol scramble control siRNA (C) and the freshly dissected Day 4 pseudopregnant mice endometrium (D). While the annexin A2 expression level in the endometrium transfected with 80 pmol annexin A2 siRNA (A) was not much lower than the controls. LE: Luminal epithelium; GE: Glandular Epithelium; SC: Stromal cells. Magnification ×400. (TIF) [file pone.0139506.s002.tif]
