# Supplementary material for: Annexin A2 Acts as an Adhesion Molecule on the Endometrial Epithelium during Implantation in Mice
Source: PLoS One. 2015 Oct 7;10(10):e0139506. doi: 10.1371/journal.pone.0139506 (PMC4596619; doi:10.1371/journal.pone.0139506)
Supplement: S1 Table — (DOCX) [file pone.0139506.s004.docx]

**Supplementary Table 1: Number of half-ERE binding sites and the variants in the promoter region of mouse Axna2 gene.**

|  | GGTCA | TGACC | GGTGA | TGGCC | GGGCA |
| --- | --- | --- | --- | --- | --- |
| A2 promotor | 8 | 7 | 4 | 5 | 7 |
|  | GGCCG | GGGTA | GGTGG | AATCA | TGACT |
|  | 0 | 0 | 5 | 0 | 7 |
